# Proteome Investigation of Rat Lungs Subjected to Ex Vivo Perfusion (EVLP)

**DOI:** 10.3390/molecules23123061

**Published:** 2018-11-22

**Authors:** Valentina Roffia, Antonella De Palma, Caterina Lonati, Dario Di Silvestre, Rossana Rossi, Marco Mantero, Stefano Gatti, Daniele Dondossola, Franco Valenza, Pierluigi Mauri, Francesco Blasi

**Affiliations:** 1 Center for Preclinical Research, Fondazione IRCCS Ca’ Granda-Ospedale Maggiore Policlinico, 20122 Milan, Italy; valentina.roffia@itb.cnr.it (V.R.); stefano.gatti@policlinico.mi.it (S.G.); 2 Proteomics and Metabolomics Unit, Institute for Biomedical Technologies (ITB-CNR), 20090 Segrate (MI), Italy; antonella.depalma@itb.cnr.it (A.D.P.); dario.disilvestre@itb.cnr.it (D.D.S.); rossana.rossi@itb.cnr.it (R.R.); 3 Department of Anesthesia and Critical Care, Fondazione IRCCS Ca’ Granda Ospedale Maggiore Policlinico, 20122 Milan, Italy; caterina.lonati@gmail.com; 4 Department of Pathophysiology and Transplantation, Università degli Studi di Milano, and Internal Medicine Department, Respiratory Unit and Adult Cystic Fibrosis Center, Fondazione IRCCS Cà Granda Ospedale Maggiore Policlinico, 20122 Milano, Italy; marco.mantero@unimi.it (M.M.); francesco.blasi@unimi.it (F.B.); 5 Surgery and Liver Transplant Center, Fondazione IRCCS Ca’ Granda-Ospedale Maggiore Policlinico, 20122 Milan, Italy; dondossola.daniele@gmail.com; 6 Department of Pathophysiology and Transplantation, University of Milan, 20122 Milan, Italy; franco.valenza@unimi.it

**Keywords:** proteomics, Ex Vivo Lung Perfusion (EVLP), nanoLC-MS/MS, transplantation

## Abstract

Ex vivo lung perfusion (EVLP) is an emerging procedure that allows organ preservation, assessment and reconditioning, increasing the number of marginal donor lungs for transplantation. However, physiological and airflow measurements are unable to unveil the molecular mechanisms responsible of EVLP beneficial effects on lung graft and monitor the proper course of the treatment. Thus, it is urgent to find specific biomarkers that possess these requirements but also accurate and reliable techniques that identify them. The purpose of this study is to give an overview on the potentiality of shotgun proteomic platforms in characterizing the status and the evolution of metabolic pathways during EVLP in order to find new potential EVLP-related biomarkers. A nanoLC-MS/MS system was applied to the proteome analysis of lung tissues from an optimized rat model in three experimental groups: native, pre- and post-EVLP. Technical and biological repeatability were evaluated and, together with clustering analysis, underlined the good quality of data produced. In-house software and bioinformatics tools allowed the label-free extraction of differentially expressed proteins among the three examined conditions and the network visualization of the pathways mainly involved. These promising findings encourage further proteomic investigations of the molecular mechanisms behind EVLP procedure.

## 1. Introduction

Ex vivo lung perfusion (EVLP) is an emerging procedure proposed for organ preservation and reconditioning which can increase the number of lungs available for transplantation without compromising its success [1]. In fact, it permits the evaluation and the treatment of lungs of doubtful function, such as ones from donors after cardiac death or from high risk donors, not only expanding the pool of possible transplants but also improving patient outcomes [2,3].

This derives from the fact that organs, previously considered unsuitable for transplantation, thanks to EVLP are now safely used and show similar or even better performances than those of lungs transplanted immediately after procurement. In these circumstances, EVLP application could represent a benefit for all donor lungs prior to implantation (so that it could be inserted in routine transplant protocols), but the evidence-based medical data collected so far are still insufficient [4] and there are different EVLP systems and application procedures adopted worldwide [5,6,7]. These weaknesses emerged on the basis of lung function measurements, but further research and tools are needed to obtain a deeper comprehension of the biological actions exerted by EVLP not only at the functional but also at the molecular level.

Accordingly, there is the need to find clinical and molecular targets that could provide evidence to recommend a specific protocol, to monitor the proper course of treatment and predict donor lung performance during EVLP procedure and after transplantation. Despite the emergence of EVLP technology, currently available methods employed for the evaluation of this procedure, are limited to airway and pulmonary measurements and therefore inadequate in assessing EVLP procedure monitoring and its molecular effects on lung graft [8]. In addition, the same respiratory diseases, for which lung transplantation is the only possible therapy, are a class of highly heterogeneous and multifactorial disorders that often make early screening, diagnosis, prognosis, disease progression, and treatment effect challenging with the diagnostic techniques applied so far. In fact, traditional methods based on reactive medicine (physiological examinations, medical history and airflow measurements) are not sufficient to fully satisfy these requirements.

All these factors contributed to the urgency in finding new specific molecular and clinical biomarkers that could be considered as objective diagnostic parameters by clinicians. In the new era of medicine [8,9], biomarkers play an essential role, because they summarize a precise biological state and provide quantifiable and comparable features that represent indicator of normal state, pathogenic processes or therapeutic response [10]. Certainly, thanks also to rapid development of next generation sequencing techniques (NGS) genomics approach is the first to be widely applied for the identification of genes as molecular biomarkers of diseases and for the selection of personalized patient therapies [11,12]. However, the genome only provides theoretical information of the protein levels while the proteome describes its actual content, which ultimately determines the phenotype. For this reason, genomic/transcriptomic investigations are recently giving way to proteomic approaches for a deepened and a more precise characterization of biological processes related to different clinical phenotypes and for the molecular monitoring of markers involved in respiratory diseases, such as COPD [13,14], cystic fibrosis [15], rhinitis [16], and the effect of anti-IgE therapy [17].

Solid organ transplantation represents an interesting challenge to test the potential of the proteomics approach and the first attempts to find new potential EVLP-related biomarkers were made by analyzing the perfusate protein expression during EVLP with multiplex immunoassays [18,19,20]. In these studies, the attention was exclusively focused on a specific class of proteins involved in inflammatory processes related to the transplant procedure.

Indeed, it is important to evaluate if these processes and other lung biological responses are effectively due to EVLP-induced changes and not to reconditioning mechanisms that could occur during the ex vivo perfusion procedure. To this end, our coauthors of Fondazione IRCCS Cà Granda Ospedale Maggiore Policlinico of Milan developed a rat model [21] that reproduces a prolonged ex vivo perfusion on uninjured lungs without the use of anti-inflammatory drugs. With gene expression studies on lung tissues and the assessment of mediators and metabolites on perfusate, they demonstrated that EVLP broadly affects the transcriptional profile and signaling pathways under monitoring [22].

In this context, the present work represents another step forward in our understanding of lung biological response under ex vivo perfusion, employing the same rat model and taking advantage of innovative proteomic technologies. In fact, advances in proteomic approaches, especially those which are mass spectrometry (MS)-based [23,24,25,26,27], could provide new tools to overcome the lack of sensitivity and specificity of traditional diagnostic techniques, and to detect more than few proteins simultaneously in a great dynamic range of concentration and in a wide variety of bio specimens [17,28].

Our main focus is to develop a shotgun label-free platform based on a nano-chip LC system combined to an Orbitrap mass spectrometer benchtop as proof of principle to characterize the complete proteome of frozen lung tissues derived from optimized EVLP rat models [21] stratified in three experimental conditions: lung biopsies collected from animals in resting conditions (native group), lung biopsies taken after death induction, in situ cold perfusion and lung procurement (pre-EVLP group), and at the end of ex vivo perfusion (post-EVLP group). An added value to this approach is given by the application of in-house software and powerful bioinformatics tools that facilitate large scale analyses, allowing the detection and validation of differentially expressed proteins as potential biomarkers of biological EVLP-induced states and the identification of metabolic pathways in which they are involved [29,30,31,32,33]. Finally, the comparison of data obtained from frozen and formalin-fixed paraffin-embedded (FFPE) tissues is also performed with the aim to point out all the potential of the platform proposed, considering the wide availability of those tissues in clinical biobanks [34].

## 2. Results

**Figure 1:**
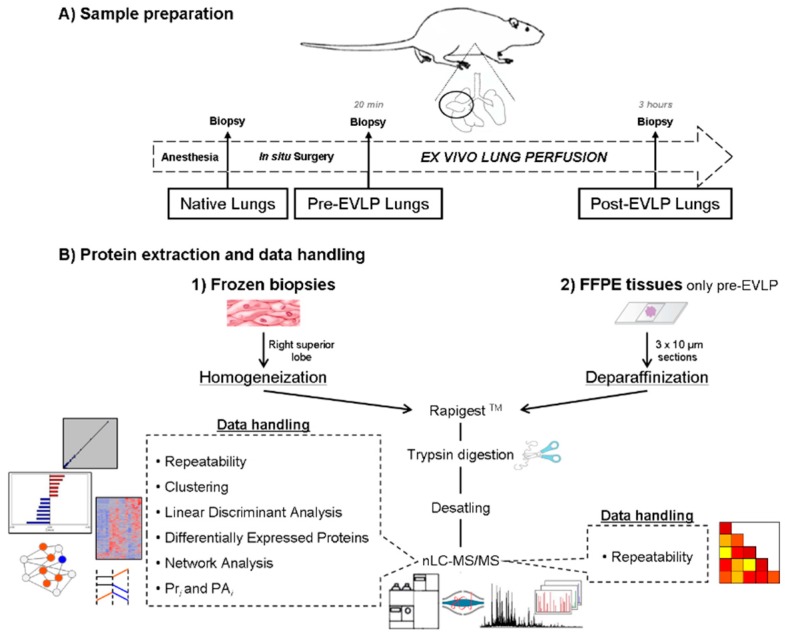
Experimental pipeline. Applied workflow for proteomic investigation of frozen and formalin-fixed paraffin-embedded (FFPE) lung tissues derived from an optimized ex vivo lung perfusion (EVLP) rat model. (**A**) Three distinct conditions (*n* = 5 for each group), native (lungs procured from animals in resting conditions), pre-EVLP (lungs subjected to in situ cold flushing and then procured), and post-EVLP (lungs subjected to in situ cold flushing, procurement and then to ex vivo perfusion/ventilation), were analyzed. (**B**) After protein extraction and digestion, the obtained peptide mixture was analyzed by a nanoLC-MS/MS system in order to allow protein identification and label-free semiquantitative data handling, such as repeatability, extraction of differentially expressed and discriminant proteins and systems biology analysis.

The application of a shotgun label-free platform, based on a nanochip LC system combined to a hybrid quadrupole Orbitrap mass spectrometer, allowed the direct analysis of lung samples obtained from a reproducible EVLP animal model and in three distinct conditions: native, pre-EVLP and post-EVLP. Specifically, each experimental condition was evaluated using five animals (biological replicates) and separately analyzed in duplicate (technical replicates), for a total of thirty nanoLC-MS/MS runs from which it was possible to identify proteins and perform label-free semiquantitative evaluation, cluster and network analyses (Figure 1).

### 2.1. Surgical Procedures and Physiological Assessments

As shown in Supplementary Table S1, mean time intervals of warm and cold ischemia were similar among animals of the post-EVLP group. Conversely, agonal phase timespan was significantly longer in the rat IE9 compared to other animals of the same experimental group (Grubb’s test, *p* < 0.05).

**Figure 2:**
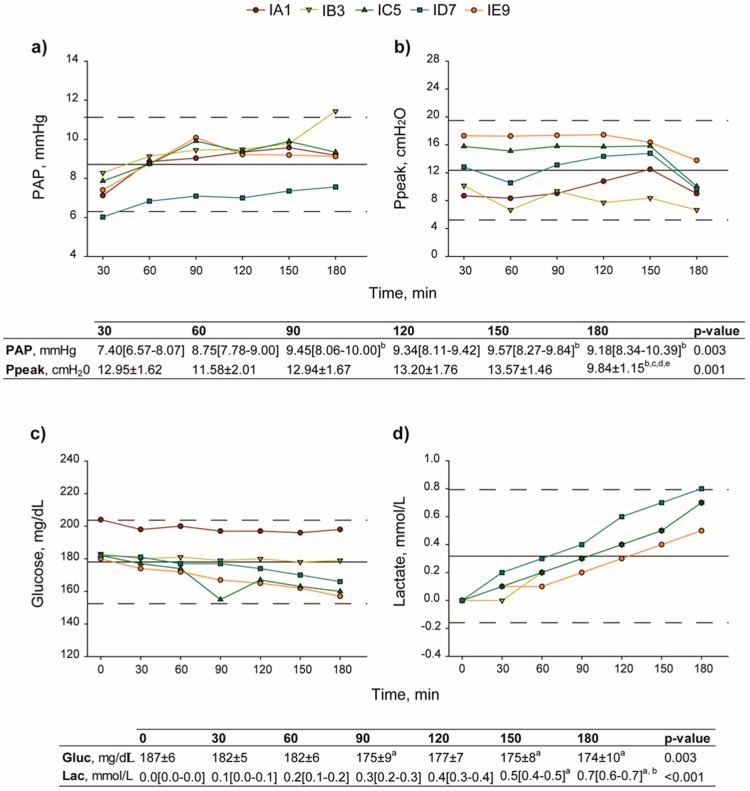
Evaluation of lung function during EVLP procedure. (**a**) PAP, significantly increased (*p* < 0.003), and (**b**) Ppeak, decreased following recruitment maneuvers (*p* < 0.001). (**c**) Concentration of glucose (from 187 ± 6 mg/dL at 0 min to 174 ± 10 at 180 min, *p* = 0.003) and (**d**) lactate (0.6–0.7 mmol/L at 180 min, *p* < 0.001) significantly changed during reperfusion. PAP, pulmonary artery pressure; Ppeak, peak inspiratory pressure. One-way repeated measures ANOVA; Tukey’s multiple comparison test. *p* < 0.05: a = vs. 0 min; b = vs. 30 min; c = vs. 90 min; d = vs. 120 min; e = vs. 150 min.

Lung functionality was evaluated through assessment of hemodynamic and respiratory parameters and measurement of the perfusate composition throughout the EVLP procedure. Pulmonary artery pressure (PAP) significantly increased (*p* < 0.003), whereas peak inspiratory pressure (Ppeak) decreased following recruitment maneuvers (*p* < 0.001). In addition, gas analysis showed that, together with a decrease in glucose concentration (*p* = 0.003), there was a rise of lactate up to 0.7 mmol/L (*p* < 0.001) (Figure 2).

Finally, the lung edema index of the Post-EVLP group was not significantly different compared to the pre-EVLP and the native groups (*p* = 0.094).

### 2.2. Protein Profiles of Frozen Tissue Lung Lobes

All of the thirty protein lists of biological and technical replicate analyses were aligned through an in-house Multidimensional Algorithm Protein Map (MAProMa) tool [29,30] and grouped by calculating, for each distinct protein, the identification frequency and the average Spectral Counts (SpC*) and Score (Score*), which are useful parameters for relative abundance protein assessment in label-free quantitative approaches. In fact, SpC represents the total number of MS/MS spectra assigned to each protein and, consequently, it reflects protein relative abundance in each analyzed condition, while Score value is another semi quantitative parameter calculated by the integration of several search engine variables. Overall, 1895 distinct proteins were identified: 1588, 1616 and 1330 for native, pre- and post-EVLP, respectively. A complete list of the identified proteins is reported in Supplementary Table S2. Figure S1a) shows the proportional Venn diagram of specific proteins for each condition. In particular, 1160 proteins were identified in all conditions, while 205 proteins were identified in both native and pre-EVLP, 75 proteins were common to pre- and post-EVLP, and 39 proteins were identified in both native and post-EVLP conditions. Finally, a total of 184, 176 and 56 distinct proteins were exclusively detected in native, pre- and post-EVLP, respectively. Using the MAProMa software it was also possible to plot each experimental condition list on a 2D virtual map, according to the theoretical Molecular Weight (MW) and Isoelectric point (pI) of identified proteins. An example is shown in Figure S1b), where the protein list of a post-EVLP sample was mapped. For each protein, a color/shape code was used according to the confidence of identification.

### 2.3. Data Handling

#### 2.3.1. Repeatability

**Figure 3:**
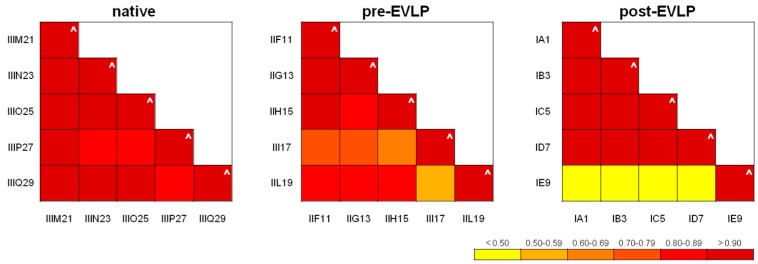
Technical and biological repeatability of proteomic analysis. Diagrams of correlation coefficient (R2) values of technical and biological replicates. Each R2 value of technical and biological replicates was assigned a color code, and the corresponding ranges associated to a chromatic scale were reported. The technical R2 value (^) was obtained comparing the spectral count (SpC) values from two runs of the same sample. Similarly, plotting the average SpC* values of two samples belonging to the same condition the biological R2 value was calculated. The numerical linear regression values (R2) for each comparison are reported in Supplementary Table S3.

**Figure 4:**
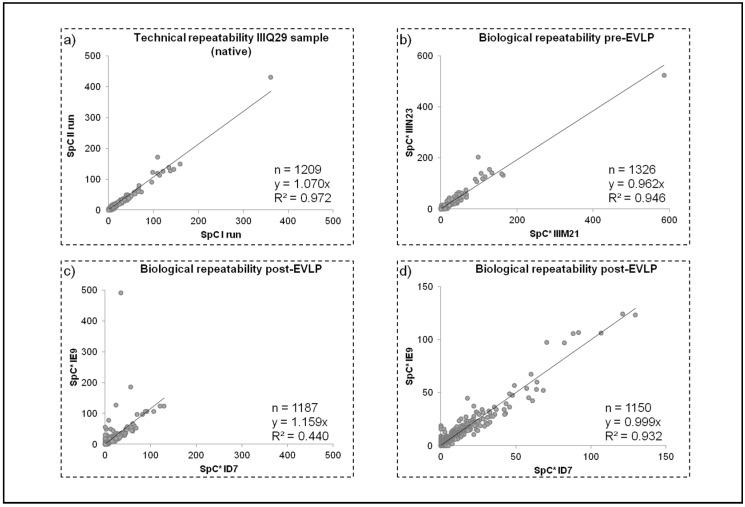
Examples of technical and biological repeatability of proteomic analysis. (**a**) Typical technical repeatability (IIIQ29 sample, native group). (**b**) Biological repeatability between two native samples (IIIM21 and IIIN23). (**c**) Biological repeatability of IE9 sample compared to ID7 (post-EVLP samples). (**d**) Biological repeatability of IE9 sample compared to ID7 without the scattered proteins (mainly plasma proteins). SpC*: Average spectral count value.

Technical and biological repeatability of the method was tested by plotting SpC values of lists previously aligned with MAProMa; specifically, each technical replicate showed linear correlation (R2) and slope close to the theoretical value of 1 (Figure 3 and Supplementary Table S3). Figure 4a reports the technical repeatability of a sample from the native group. Similarly, the biological repeatability was verified (Supplementary Table S3 and Figure 3). As shown in Figure 4b, the average SpC of sample IIIM21 (X axis) was compared with that of IIIN23 (Y axis), both belonging to the post-EVLP condition: R2 = 0.946 and y = 0.962. These findings demonstrated the good repeatability of the proteomic approach adopted, because the reduced oscillations of SpC values are a robust starting point for subsequently label-free investigations on protein profiles. Furthermore, similar results were also reported for all the analyzed samples (data not shown), except for sample IE9 (belonging to the post-EVLP condition) which shows a lower biological repeatability compared to the other samples in the same condition. An example of low biological repeatability with sample ID7 was reported in Figure 4c. For this reason sample, IE9 was further investigated and only 30 proteins (out of 1187) resulted significantly scattered in IE9 (compared to the other post-EVLP samples); in particular, these nonlinear proteins were mainly related to plasma proteins. Of note, deleting the plasma related proteins identified in the IE9 sample the correlation with the other post-EVLP samples improved (Figure 4d). For these reasons, the IE9 sample was taken into account for label-free quantitation and network analysis. The proteomics finding in the IE9 sample were in agreement with its different agonal phase as reported in Section 2.1. The different behaviors of the IE9 sample (both biological and molecular) could be correlated to possible lesions due to the supplemental manipulation (double cannulation required) that occurred to IE9 during the surgical procedure.

#### 2.3.2. Clustering and Linear Discriminant Analyses

**Figure 5:**
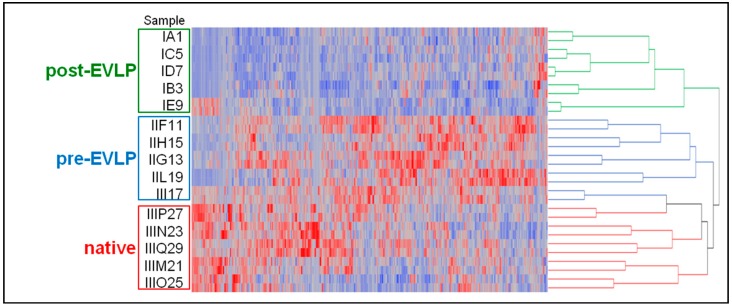
Hierarchical clustering obtained by Linear Discriminant Analysis (LDA). Using the average SpC values from all conditions, the post-EVLP samples were correctly segregated and 451 descriptor proteins by LDA were identified. Of note, IE9 sample, although remaining within the correct subgroup, is localized on a separate branch. The complete list of descriptor proteins extracted by LDA is reported in Supplementary Table S4. filtering criteria.

Protein lists obtained by the proteomics analyses of the three experimental conditions analyzed were processed by Linear Discriminant Analysis (LDA) [35] and 451 proteins with F ratio > 3.4 and *p*-value < 0.05 were selected as descriptors of features able to correctly discriminate the three groups (Supplementary Table S4). An oriented dendogram was obtained using all the average SpC protein values of the fifteen animals analyzed. As shown in Figure 5, all post-EVLP samples resulted segregated into a group and separated from native and pre-EVLP samples. Of note, observing the post-EVLP grouping more in detail, the IE9 sample, while remaining within the same cluster, shows a slightly different trend than the other animals in the group and is localized on a separate branch, much closer to the pre-EVLP and native ones.

#### 2.3.3. Differential Analysis

Independently from cluster analysis, a label-free evaluation of differentially expressed proteins was also performed using two parameters available in MAProMa software and comparing the average SpC values obtained for each identified protein in the three examined conditions. These parameters are DAve (Differential Average) and DCI (Differential Coefficient Index) representing the ratio and the confidence in differential expression, respectively, of a protein between two samples. They allowed the identification of 239 distinct differentially expressed proteins (DEP), using the filters described in Material and Methods section (Section 4.4.3). In particular, 31 and 77 proteins resulted respectively down- and upregulated in pre-EVLP compared to native. On the other hand, 180 resulted more abundant in pre-EVLP compared to post-EVLP, and only three proteins were upregulated in post-EVLP. Finally, 135 and 11 were down- and upregulated, respectively, in post-EVLP compared to native. Noteworthy, the combination of the data obtained by the differential evaluation with LDA and DEP analysis allowed the identification of 499 proteins, among which, 190 distinct proteins resulted common to the two methods applied. In fact, about 80% of DEP were confirmed by LDA. The complete list of differentially expressed proteins is reported in Supplementary Table S4, where proteins derived from the same gene were grouped.

### 2.4. Network Analysis

Proteins selected by LDA and DEP analyses were used to reconstruct a Protein–Protein Interaction (PPI) network of *Rattus norvegicus* involving 487 nodes and 7806 interactions for the investigation of the functional relationships among groups of proteins identified in our proteomic approach. Considered nodes were grouped into 25 distinct subnetworks based on their function and/or subcellular localization. These subnetworks were grouped into five distinct main groups: extracellular matrix, translation, cytoskeleton, cell cycle, and metabolism. The proteins displayed on the network are represented by a chromatic scale that reflects the relative abundance (SpC) of their expression. By means of Cytoscape [36] it is also possible to upload to the same metabolic network the results derived from the label-free differential analysis of pairwise comparison: native vs. pre-EVLP (Supplementary Figure S2a), pre-EVLP vs. post-EVLP (Supplementary Figure S2b), and native vs. post-EVLP (Supplementary Figure S2c). The majority of subnetworks resulted mainly up- and downregulated in pre- and post-EVLP, respectively, compared to the native condition. Of note, some subnetworks, such as redox, PDIA (Protein Folding Drives Disulfide Formation), actin- and myosin-related proteins and vesicle-mediated transport seem unchanged in native vs. post-EVLP comparison; on the contrary, these subnetworks resulted up regulated in pre-EVLP.

**Figure 6:**
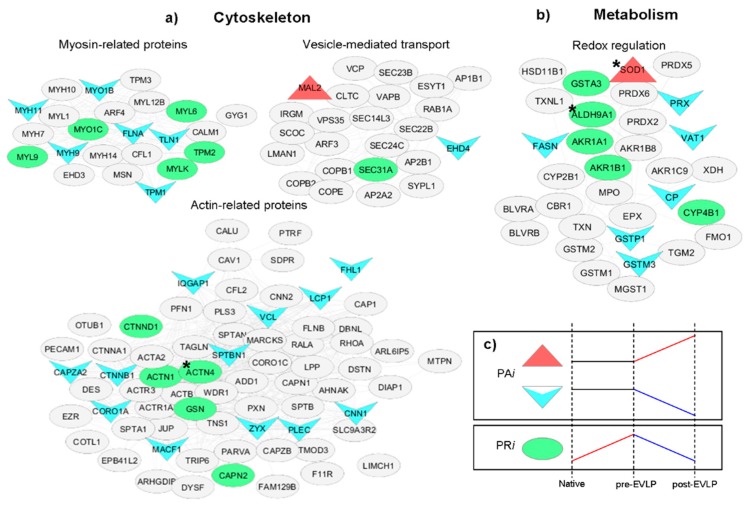
Main pathways involved in the proteome remodeling induced by the EVLP procedure. Enriched subnetworks based on recovered and activated proteins after ANOVA (<0.05) and Tukey’s test (<0.05; * <0.08). These statistically significant proteins were previously extracted using an unbiased procedure that evaluated simultaneously the changes in the proteome in the three conditions: native, pre-EVLP, and post-EVLP. Recovered proteins, marked as a green/circle, were extracted using the Proteome Remodeling index (PRi); while activated proteins marked as red/triangle and blue/arrow for up- and downregulated in post-EVLP, respectively, were extracted using Proteome Activated index (PAi). The application of PRi and PAi filters highlights an enrichment in (**a**) vesicle-mediated transport, myosin-, and actin-related proteins, belonging to cytoskeleton and (**b**) redox regulation, belonging to metabolism. The blank nodes identify the protein descriptors resulted outside the applied PRi and PAi filters. (**c**) Summary of considered trends of remodeled proteins. The complete lists of PRi and PAi extracted proteins are reported in Supplementary Tables S5 and S6.

To further investigate more the modifications of proteome in response to the EVLP treatment, it was calculated for each protein its variations (modules) due to perturbation (native vs. pre-EVLP) and treatment (pre-EVLP vs. post-EVLP). Theoretically, if the two modules present similar values but they have opposite sign, the protein recovers its level to the reference (in our case native condition). The extraction of so-called “recovered proteins” was simplified by calculating the Proteome Remodeling index (PRi) for each protein using an unbiased procedure. In this way, we extracted 104 proteins, and 32 had a statistically significant result (ANOVA ≤ 0.05, Supplementary Table S5). Proteome Activated index (PAi) was calculated using baseline and treatment modules to extract proteins which unchanged between native and pre-EVLP conditions (perturbation module), but resulted to be activated or inhibited (up- or downregulated, respectively) with treatment (post-EVLP condition). In this case 168 proteins were found, of which 67 resulted statistically significant by ANOVA (≤0.05) (Supplementary Table S6). Of note, only two proteins resulted increased with the EVLP treatment. These extracted proteins were uploaded in the PPI network previously prepared and four subnetworks resulted to be enriched (Figure 6 and Figure S3): myosin-related proteins, actin-related proteins, and vesicle-mediated transport (belonging to cytoskeleton), and redox-processes (metabolism). Similar results were obtained by means of ln[FoldChange] (Supplementary Tables S5 and S6).

### 2.5. Comparison between Frozen and FFPE Tissue Lung Lobes

**Figure 7:**
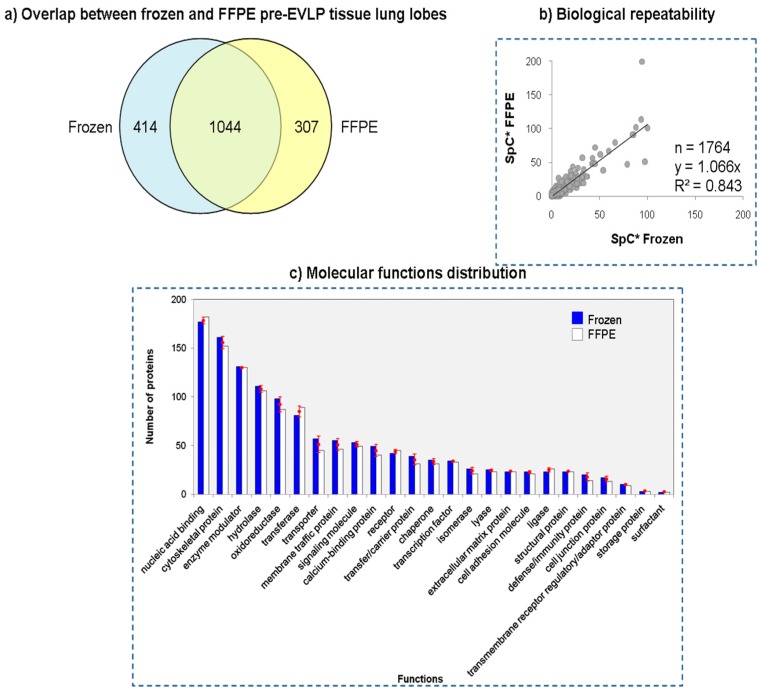
Comparison between frozen and FFPE pre-EVLP lung lobes. (**a**) Protein overlapping between frozen and FFPE in three pre-EVLP analyzed samples (IIL11, IIG13 and IIH15). (**b**) Plot of frozen vs。 FFPE samples using normalized average spectral count (SpC*) values. (**c**) Distribution of proteins between the frozen and FFPE according to their molecular function using Protein ANalysis THrough Evolutionary Relationships (PANTHER) classification system. For each molecular function, reported as percentage of proteins for the two tissues (blue/solid colour for frozen and blank for FFPE datasets), the difference frozen vs. FFPE was <0.9%.

Shotgun proteomics approach was also employed to compare frozen and FFPE lung tissues. In particular, three FFPE samples from the pre-EVLP condition were analyzed. Using MAProMa software the three FFPE resulting proteomes were aligned with the corresponding frozen ones. The resulting twelve nanoLC-MS/MS runs allowed the identification of 1764 distinct proteins: 1458 and 1351 for frozen and FFPE, respectively (Table S7). As reported in Figure 7a more than 77% (1044) of FFPE proteins were confirmed in frozen specimens. Plotting the normalized average SpC values between the two datasets both R2 and slope were close to the theoretical value of 1 (Figure 7b). Finally, through the Protein ANalysis THrough Evolutionary Relationships (PANTHER) classification system [33] it was possible to classify proteins based on their molecular function. As shown in Figure 7c the distribution of proteins between the FFPE and frozen tissues was nearly identical, suggesting that the differences in proteins observed in the two datasets were not biased toward a specific molecular function.

## 3. Discussion

Here, we employed a rat model and recent advances in MS-based proteomics to generate a proteomic system biology view of the EVLP procedure. The EVLP procedure cannot be solely evaluated on the basis of physiological measurements. In fact, analyses at the molecular level are mandatory to find potential protein markers for a better understanding of mechanisms by which EVLP is ruled [18,19,20]. In light of this our approach represents a first attempt in this direction, with the intent to develop a shotgun label-free platform which could be helpful in extracting informative features from complex biospecimens in large scale analyses useful to correlate molecular expressions to biological processes. In particular, a nanoLC system based on chip technology was combined to a next-generation mass spectrometer with improved resolution and accurate mass detection in order to investigate protein extracts obtained from frozen lung tissues in three experimental groups (native, pre-EVLP and post-EVLP). This high throughput system was completely automatized and allowed the simultaneous identification of thousands of proteins with a wide range of concentrations and without limits of pI and MW [17,28] (see Figure 1).

Moreover, the large amount of data produced were properly processed with dedicated, commercial or in-house, bioinformatic tools using parameters provided by the employed search engines [29,30,31,32,33]. In our case, data handling was greatly simplified by means of MAProMa software [29,30] since it allowed the automatic alignment of different protein lists, presenting the results in a user-friendly format and highlighting differentially expressed proteins. In addition, data dimensional reduction with support vector machines was also applied to extract informative features useful for the correct classification and sample grouping using only information correlated to them and their relationships [35]. In this respect, the hierarchical clustering analysis performed by LDA and conducted on the fifteen average protein lists obtained by the three examined conditions, permitted to discern the differences among them and to correctly group samples in the three distinct categories (Figure 5). These findings indicate the high reproducibility and statistical reliability of the proposed approach. An additional confirmation derived from the technical and biological repeatability results obtained comparing SpC values of protein lists of the same sample injected in replicate and of different animals belonging to the same experimental group (Figure 3). In this case, our platform was able to point out differences in the IE9 sample with respect to the other biological replicates of the post-EVLP group. Specifically, the IE9 sample showed a very low biological repeatability in all the comparisons with animals of the same experimental group (Figure 3c). With the aim of further understanding the IE9 molecular variability, the protein profiles of this animal and its biological replicates were investigated. The results of differential analysis showed a similar pattern of IE9 with the other post-EVLP animals except for only a few proteins that resulted up regulated and essentially related to blood components. These findings were in agreement with the longer agonal phase of this animal herein reported and could be correlated to possible lesions due to the double cannulation incurred during the surgical procedure. However, this did not compromise the final functional outcome of the animal, which was not excluded from the study, but with its unique profile revealed the potentiality of our platform as confirmed by clustering analysis (Figure 5). In contrast to the inability of functional measurements to detect even small differences, clustering correctly maintained IE9 within the post-EVLP group but underlined its incomplete alignment with the other components of the same group, providing in this way a deeper and more accurate data evaluation strategy.

Another interesting feature that could be supported by our approach is the possibility to conduct a label-free quantitative investigation for the identification of potential biomarkers. Given the increasing popularity of EVLP treatment, the search for new molecular targets which could be employed in the future as objective parameters to recommend a protocol over others, to monitor the entire procedure and to predict lung performance is urgent. With this in mind, our study represents a first effort in this direction, as far as we know. In particular, LDA analysis showed the post-EVLP group completely segregated from the pre-EVLP and native conditions and provided the selection of 451 proteins whose trends were able to discriminate the three groups (Figure 5). Using an independent methodology, supplied by the application of DAve and DCI parameters included in MAProMa software [29,30], 246 distinct differentially expressed proteins were found with an overlap of 80% with those previously extracted by LDA.

At this point, the differentially expressed proteins resulting from both these computational tools were uploaded in a network built using Cytoscape [32] and with which it was possible to visualize protein–protein interactions and metabolic pathways mainly involved. As reported in the Results section, the differentially expressed proteins were network visualizable with a color code that represented the DAve values resulting by the pairwise comparison of the three conditions (Supplementary Figure S2a–c). What emerged from a first observation was that EVLP treatment involved a downregulation of a great number of proteins reported in the metabolic pathways. If, from a functional point of view, both EVLP and native lungs seemed to achieve the same results in term of graft acceptability criteria, the study of molecular profiles suggests that this take place in different ways with the intervention of different pathways. Certainly, the experimental data permitted to exclude that this phenomenon was attributable to sample degradation due to a different processing time that the EVLP treatment required. In fact, the total number of identified proteins in the three conditions presented similar orders of magnitude and only 13% of the distinct identified proteins showed a differential expression (Supplementary Table S1).

Moreover, by means of proteome remodeling indexes, PR*i* and PA*i*, proteomic changes were simultaneously evaluated in pre-EVLP and in post-EVLP, where proteome expression in the latter seems to return to the reference condition (native group). After EVLP treatment, approximately 32 proteins resulted statistically significant in rebalancing the proteome to the reference condition of native lungs. Based on these findings it was possible to speculate that restored proteins might be correlated to the reconditioning effect exerted by EVLP. Of note, these proteins were always down regulated in post-EVLP compared to pre-EVLP; specifically, they resulted mainly involved in cytoskeleton (vesicle-mediated transport and myosin- and actin-related proteins) and in redox regulation (metabolism) (Figure 6). Another interesting class of proteins extracted by calculating the Proteome Activated index (PA*i*) did not show differential expression between native and pre-EVLP conditions, but increased or decreased their abundance after EVLP treatment. In particular only Mal2 and SOD1 increased their level, while 65 proteins decreased their level in post-EVLP compared to both native and pre-EVLP groups. Similarly to PR*i*-extracted proteins, those PA*i*-extracted resulted being mainly involved in cytoskeleton (vesicle-mediated transport and myosin- and actin-related proteins) and in redox regulation (metabolism).

To better understand these data and hypothesize possible mechanisms that govern the activation or suppression of metabolic pathways, we need to keep in mind what happens at a biochemical level during ex vivo lung perfusion. During the entire EVLP procedure, the lungs are subjected to a first phase of oxygen breakdown, for cold flushing and recruitment of organ (cold ischemia), and to a subsequent reperfusion by ex vivo perfusion/ventilation. The reduction of O_2_ causes alterations in cellular metabolism and the switch from aerobic to anaerobic metabolism [37] with the subsequent reduction of intracellular ATP. Alteration of cell pH and generation of reactive oxygen species (ROS) [38,39] due to increase of Na^+^ [40,41] and Ca^2+^ ions [42,43] are significant events that also occur. In addition, changes in cytoskeleton composition are signaled in endothelial cells with the activation of adhesion proteins [44] and others one involved in the maintenance of flexibility and adaptability of cytoskeleton during hypoxia [45].

As stated before, all these effects attributable to EVLP on different biomolecular pathways are easily observable in networks built with proteins highlighted for their differential expression and extracted by MAProMa, LDA and remodeling indexes. In particular, it is interesting to note that, among upregulated proteins in the post-EVLP condition, there are some belonging to the extracellular matrix (ECM), constituents of the basement membrane, and involved in inflammatory process and its regulation. Their secretion is stimulated by IL-1β and TNF-α [46,47] in order to repair the basement membrane, regenerating ECM around pulmonary fibroblasts and reconstituting epithelial tissue integrity after lung injury [47]. Of note, our coauthors of the Fondazione IRCCS Cà Granda Ospedale Maggiore Policlinico of Milan, in a work just published [22] and based on studies of gene expression on the same animal model samples, observed that IL-1β and TNF-α resulted upregulated in EVLP, due to lung injury resulting in the induction of ischemia by the surgical procedure, and activated the NF-kB complex. The occurrence of an inflammatory response together with other alterations at the proteomic level in lungs, in a rat model designed without the use of anti-inflammatory drugs that could falsify results, is to be considered another small step towards the discovery of molecular mechanisms modulated by EVLP.

If on the one hand during riperfusion the activation of inflammatory processes leads to the expression of pro-inflammatory mediators (like IL-1β and TNF-α) [48,49] on the other hand stimulates the release of reactive oxygen species (ROS) [50,51,52]. Together with the involvement of redox regulation and heat shock response in our networks, the observed SOD1 increase during the EVLP treatment has just the purpose to decrease ROS levels and to neutralize ROS-dependent damage. This seems to perform a protective action to contrast the inflammatory effect triggered by the procedure itself and raises the possibility that EVLP donor lungs could be less immunogenic than standard ones. The regulation of described proteins can be considered in all respects an integral part of the mechanism of action of the EVLP treatment and it may explain at least in part its efficacy in restoring lung function, or, as suggested by Lonati et al. [22], the resistance to damage caused by ischemia/reperfusion occurrence after transplantation. Thus, these findings add new key information to the mechanism behind potential EVLP beneficial effects and encourage additional research to implement EVLP therapeutic interventions. If this is the case, unveiling the modes of action of EVLP on lung phenotype and its monitoring at the molecular level, may be crucial not only for improving the transplant procedure but also in providing a powerful tool in translational medicine and in the advancement of pulmonary physiology. In addition, the present study can provide useful information to implement ex vivo perfusion procedure of other solid organs.

In order to give a more complete view of our workflow potential, we performed a preliminary proteomic characterization of FFPE tissues. In particular, we analyzed three FFPE samples belonging to the pre-EVLP condition in order to compare their protein profiles with corresponding frozen tissues. Despite the low overlap between FFPE and frozen datasets (around 60%), R^2^ and slope values plotting the normalized average SpC values of the two tissues resulted being close to 1 (Figure 7). This suggests that the differences in the two pools were only apparent and probably related to the effects of fixation. In fact, as proof of this, the molecular function distribution of the identified proteins between frozen and FFPE tissues was nearly identical and not biased toward a specific molecular function. The last finding opens up the possibility to work with FFPE tissues and to connect proteomic outputs with information on clinical outcomes.

Obviously, these results are still to be considered as preliminary and must be thoroughly investigated; however, these results offer the possibility to generate with a proteomic platform an overview of what happens at the molecular level during the entire transplant procedure with the ability to track the pattern of target proteins. In this regard, our approach facilitates the extraction of signature proteins from large datasets and their distribution in multiple pathways and quantitative traceability without the need of labeling, make these proteins ideal targets in the biomarker validation phase. In fact, the ongoing developments of MS-based proteomic technologies combined to increasingly powerful bioinformatics tools are accelerating their employment as a valid alternative to traditional assay methods for a number of features including: increased sensitivity, cost containment and simultaneous recording of multiple biomarkers.

Of course, our study represents a proof of principle with which we want to prove the potential of a shotgun proteomic platform in these investigations. However, animal model and sample size limit drawing any further conclusions on proteins and pathways discussed above. In the near future, it will be necessary to increase the cohort of study and translate to human specimens the experimental research in order to confirm the findings derived from animal models and to overcome possible drawbacks due to the incomplete overlap of data.

Another interesting aspect will be represented by the possibility of using other biospecimens, in addition to lung tissues, for minimizing invasiveness without compromising diagnostic accuracy. In this respect, exosomes and extracellular vesicles (EVs) are attracting great interest. As reported by Vallabhajosyula et al. [53] in a study on the perfusate from EVLP lungs, understanding EVs biology may have important diagnostic and therapeutic implications in pulmonary medicine, expanding even more the potentiality of the EVLP model.

## 4. Materials and Methods 

### 4.1. Experimental Proteomic Pipeline

Figure 1 shows a schematic overview of the protocol adopted to prepare frozen and FFPE tissue lung lobs prior to the nanoLC-MS/MS analysis. The protocol consists of different parts that will be described in detail below.

### 4.2. Surgical Procedure and Frozen and FFPE Tissue Collection

#### 4.2.1. Animals and Experimental Groups

The experiments were performed in strict accordance with the recommendations in the Guide for the Care and Use of Laboratory Animals of the National Institutes of Health, at the Center for Preclinical Investigation, Fondazione IRCCS Ca’ Granda Ospedale Maggiore Policlinico, Milan, Italy. The experimental protocol was approved by the Italian Institute of Health (permit number: 1082-15).

Adult Sprague–Dawley male rats (Charles River, Calco, Lecco, Italy) weighing 270 to 330 g were housed in a ventilated cage system (Tecniplast S.p.A., Varese, Italy) at 22 ± 1 °C, 55 ± 5% humidity, on a 12 h dark/light cycle, and were allowed free access to rat chow feed and water ad libitum.

Rat were randomly assigned to one of the following experimental groups (*n* = 5 in each group). Following sacrifice of rats under general anesthesia, lungs were subjected to in situ cold flushing and were then retrieved (pre-EVLP group). In separate experiments, after in situ cold flushing, isolated lungs underwent ex vivo perfusion/ventilation for 180 min (post-EVLP group). Finally, in a third group lung biopsies were taken from animals in resting conditions (native group). Samples of perfusion fluid were collected at time intervals throughout EVLP procedure for gas analysis. Tissue biopsies were harvested to investigate lung edema and proteome. Specifically, right superior lobes were used for lung edema evaluation, right inferior lobes were snap-frozen and stored at −80 °C for proteomic analysis, and median lobes were added to 4% formalin at room temperature.

#### 4.2.2. Ex Vivo Lung Perfusion (EVLP)

Anesthesia, in situ flushing and lung procurement were performed according to our previous work. Briefly, after sacrifice, lungs were flushed with ice-cold Perfadex^®^ (XVIVO Perfusion AB, Gotebörg, Sweden). Thereafter, the heart-lungs block was procured and connected to an isolated lung perfusion system (Hugo Sachs Elektronik, Harvard Apparatus, GmbH, March-Hugsstetten, Germany) primed with in-house acellular solution with extracellular electrolyte composition [21]. No treatment with corticosteroids or other anti-inflammatory drugs was performed.

The protocol for rat ex vivo lung perfusion included a reperfusion phase (from 0 to 40 min) during which the lungs were gradually re-warmed, ventilated and perfused in order to avoid hemodynamic stress, and a steady-state phase (from 40 to 180 min). Specifically, initial pulmonary artery flow was set at 20% of target value and was then augmented over 40 min. Lung temperature was enhanced up to 37 °C in a 25 min time-span. Volume-controlled ventilation was started when the lungs reached a normothermic state, with respiratory rate of 35 bpm and pulmonary end expiratory pressure (PEEP) at 3 cmH2O. Initial tidal volume was set at 5 ml/kg and was increased up to 7 ml/kg in 10 min.

#### 4.2.3. Lung Function

- Pulmonary artery and airway pressure assessment.
  PAP and Ppeak were continuously recorded, and digitally stored via an amplifier (data acquisition software Colligo Elekton, Milan, Italy).
- Gas analysis and determination of glucose and lactate concentration.
  Gas analysis, glucose, and lactate concentration evaluation were performed on perfusate samples using a gas analyzer (ABL 800 FLEX, A. De Mori Strumenti, Milan, Italy).
- Lung edema index.
  At the end of the procedure, the right superior lobe was weighed (KERN & SOHN GmbH, Balingen, Germany), before (wet) and after (dry) storage at 50 °C for 24 h (LTE Scientific, Oldham, UK). Wet-to-dry ratio (*W*/*D*) was then calculated and used as index for pulmonary edema.

### 4.3. Protein Extraction

#### 4.3.1. Frozen Lobes

For proteomic analysis of frozen samples, each lobe was suspended in 300 µL 0.1 M H_4_HCO_3_ pH 7.9 buffer with 10% acetonitrile (Sigma-Aldrich Inc., St Louis, MO, USA) and homogenized in ice. The protein concentration was assayed using dotMETRIC^TM^ (G-Biosciences, St. Louis, MO, USA) and the membrane proteins were solubilized by adding Rapigest^TM^ SF reagent (Waters Co, Milford, MA, USA) at the final concentration of 0.33% (*w*/*v*). The resulting suspensions were incubated under stirring at 100 °C for 5 min. The digestion was carried out on 50 ± 0.5 µg proteins of each sample by adding Sequencing Grade Modified Trypsin (Promega Inc., Madison, WI, USA) at an enzyme/substrate ratio of 1:50 (*w*/*w*) overnight at 37 °C. An additional aliquot of 2 µg of trypsin (1:25 *w*/*w*) was added in the morning, and the digestion continued for 4 h. Moreover, the addition of 0.5% Trifluoroacetic acid (TFA) (Sigma-Aldrich Inc., St Louis, MO, USA) stopped the enzymatic reaction, and a subsequent incubation at 37 °C for 45 min completed the RapiGest acid hydrolysis [54]. The water immiscible degradation products were removed by centrifugation at 13,000 rpm for 10 min. Finally, the tryptic digest mixture was desalted using PepClean^TM^ C-18 spin column (Pierce, Rockford, Il, USA) and was resuspended in 0.1% formic acid (Sigma-Aldrich Inc., St. Louis, MO, USA) in water. Water was deionized by passing through a Direct Q (Millipore, Bedford, MA, USA).

#### 4.3.2. Formalin-Fixed Paraffin Embedded (FFPE) tissues

Paraffin-embedded tissue sections were dewaxed by means of xylene deparaffinization. Briefly, each lung biopsy was cut up to obtain five 10 µm-sections from the same block. Slices were placed in a 1.5 mL collection tube and added to xylene (Sigma-Aldrich Inc., St Louis, MO, USA). Following 10 min-incubation at room temperature, samples were centrifuged at 13,000 rpm for 2 min. Supernatants were discarded and pellets underwent two additional xylene washes. Next, samples were rehydrated using increasing ethanol dilutions (100%, 96%, and 70% ethanol, Sigma-Aldrich Inc., St Louis, MO, USA). Dry pellets were stored at −80 °C.

The dewaxed tissues from 3 pre-EVLP samples (IIF11, IIG13 and IIH15), were solubilized in Rapigest^TM^ SF reagent at the final concentration of 0.25% (*w*/*v*) and incubated under stirring, first at 100 °C for 20 min and then sequentially at 80 °C for 2 h. Then the protein concentration was assayed using dotMETRIC^TM^, and 24 ± 2.1 µg of proteins from each sample was digested by Sequencing Grade Modified Trypsin at an enzyme/substrate ratio of 1:25 (*w*/*w*) overnight at 37 °C. An additional aliquot of 0.5 µg of trypsin (1:12,5 *w*/*w*) was added in the morning, and the digestion continued for 4 h. The enzymatic digestion was stopped with the addition of 0.5% TFA, and a subsequent incubation at 37 °C for 45 min completed the RapiGest acid hydrolysis [54]. The water immiscible degradation products were removed by centrifugation at 13,000 rpm for 10 min. Finally, the tryptic digest mixture was desalted using PepClean^TM^ C-18 spin column and was resuspended in 0.1% formic acid in water.

### 4.4. Proteomic Analysis

#### 4.4.1. Liquid Chromatography

Trypsin digested mixture were analyzed using Eksigent nanoLC-Ultra^®^ 2D System (Eksigent, part of AB SCIEX, Dublin, CA, USA) combined with cHiPLC^®^-nanoflex system (Eksigent) in trap-elute mode. Briefly, samples were first loaded on the cHiPLC trap (200 µm × 500 µm ChromXP C18-CL, 3 µm, 120 Å) and washed with the loading pump running in isocratic mode with 0.1% formic acid in water for 10 min at a flow rate of 3 µL/min. The automatic switching of cHiPLC ten-port valve then eluted the trapped mixture on a nano cHiPLC column (75 µm × 15 cm ChromXP C18-CL, 3 µm, 120 Å) through a 65 minute gradient of 5 to 40% of eluent B (eluent A, 0.1% formic acid in water; eluent B, 0.1% formic acid in acetonitrile) at a flow rate of 300 nL/min. Trap and column were maintained at 35 °C for retention time stability.

#### 4.4.2. Mass Spectrometry

MS spectra were acquired using a QExactive mass spectrometer (Thermo Fisher Scientific, San Jose, CA, USA), equipped with an EASY-Spray ion source (Thermo Fisher Scientific). Easy spray was achieved using an EASY-Spray Emitter (Dionex Benelux BV, Amsterdam, The Netherlands) (nanoflow 7 µm ID Transfer Line 20 µm × 50 cm) held to 2.1 kV, while the ion transfer capillary was held at 220 °C. Full mass spectra were recorded in positive ion mode over a 400 to 1600 *m/z* range with a resolution setting of 70,000 FWHM (Full Width at Half Maximum) (@ *m*/*z* 200) with 1 microscan per second. Each full scan was followed by 7 MS/MS events, acquired at a resolution of 17,500 FWHM, sequentially generated in a data dependent manner on the top seven most abundant isotope patterns with charge ≥2, selected with an isolation window of 2 *m*/*z* from the survey scan, fragmented by higher energy collisional dissociation (HCD) with normalized collision energies of 30 and dynamically excluded for 30 sec. The maximum ion injection times for the survey scan and the MS/MS scans were 50 and 200 ms and the ion target values were set to 10^6^ and 10^5^, respectively.

The MS data have been deposited to the ProteomeXchange Consortium via the PRIDE [55] partner repository with the dataset identifier PXD006692.

#### 4.4.3. Data Handling

All data generated were processed using the SEQUEST search engine [56] of BioWorks platform (version 3.3.1, University of Washington, licensed to ThermoFinnigan Corp., San Jose, CA, USA). The experimental MS/MS spectra were correlated to tryptic peptide sequences by comparison with theoretical MS/MS spectra obtained by in silico digestion of the Rattus norvegicus protein database (about 79609 entries), downloaded (January 2014) from the National Centre for Biotechnology Information (NCBI) website (www.ncbi.nlm.nih.gov). This allowed the identification of peptide sequences and related proteins. The confidence of protein identification, particularly when using data from a single peptide, was assured by applying stringent filtering criteria: the MS/MS tolerance was set to 1.00 Da; the precursor ion tolerance was set to 50 ppm (part-per million); and the intensity threshold was set to 100. Moreover, searches were performed with no enzyme. Finally, to assign a final score to the proteins, the SEQUEST output data were filtered by setting the peptide probability to 1 × 10^−3^, the chosen minimum correlation score values (Xcorr) were 1.5, 2.0, 2.5, and 3.0 for single-, double-, triple-, and quadruple-charged ions, respectively, and the consensus score higher than 10 (FDR < 0.01). The output data obtained from the SEQUEST software were aligned, compared and grouped by calculating the average Spectral Count (SpC*) and Score values for each distinct protein using an *in-house* algorithm, namely, the Multidimensional Algorithm Protein Map (MAProMa) [29,30].

#### 4.4.4. Label-Free Differential Analysis

Differential analysis was performed by comparing the average Spectral count (SpC*) values of each protein, obtained from native, pre-EVLP and post-EVLP lungs, using Dave and DCI algorithm inserted in the MAProMa software. DAve is an index of the relative ratio between the two compared protein lists and DCI is an index to evaluate the confidence of DAve. Most confident uprepresented proteins in lung tissues showed a DAve ≥ +0.2 and a DCI ≥ +10; most confident downrepresented proteins showed a DAve ≤ −0.2 and DCI ≤ −10.

#### 4.4.5. Hierarchical Clustering and Linear Discriminant Analysis

Biological replicates were evaluated using an unsupervised learning method, such as hierarchical clustering (HC) [57] using in-house R-scripts, which was based on the XlsReadWrite, clue, and clValid libraries (http://cran.r-project.org). In particular, the Euclidean distance metric was applied, and an Agglomerative Coefficient was calculated (Agglomerative Nesting—AGNES). AGNES, which as additional information, yields the AC that measures the amount of clustering structures found. AC is a dimensionless quantity, which could vary between 0 (bad structure) and 1 (very clear structuring). In order to perform multivariate analysis, all protein lists were processed using Linear Discriminant Analysis (LDA) [35]. This analysis was applied by using a common covariance matrix for all the groups and the Mahalanobis distance [58] from each point to each group’s multivariate mean (proteins derived from the same gene were grouped). The identifying features (proteins) that discriminated the analyzed samples were considered to be those with the largest F ratio (>3.4) and the smallest *p*-value (<0.05). Specifically, the F ratio represented the model mean square divided by the error mean square, whereas the p-value indicated the probability of obtaining an F value greater than that calculated if, in reality, there was no difference between the population group means.

#### 4.4.6. Network Analysis

Starting from the list of experimentally identified proteins, the corresponding Rattus norvegicus Protein-Protein Interaction (PPI) network was extracted. By means of a Cytoscape plug-in, STRING 8 database (http://string.embl.de/) [36], known interactions were retrieved from several databases such as Prolink, DIP, KEGG, BIND and others. All types of interactions were retrieved from each repository without applying a *p*-value threshold. Protein–DNA, protein–RNA, protein–metabolite, and protein–drug interactions, if present in the data sets, were removed, as were duplicates and self-interactions. PPI were examined using Cytoscape 3.2.1 (http://www.Cytoscape.org) [32] by applying a score >0.15. Protein–DNA, protein–RNA, protein–metabolite, and protein–drug interactions, if present in the data sets, were removed, as were duplicates and self-interactions. PPI were processed using Cytoscape 3.2.1 (http://www.cytoscape.org/) [32]. Proteins that were not mapped or were mapped as isolated components were not considered in the analysis. Bingo 2.44 [59], a Cytoscape plug-in, was used to emphasize subnetworks based on functionally organized GO terms, and the MCODE plug-in [60] was used to cluster subnetworks based on their topology and, specifically, by considering densely connected regions. Further selection of nodes was made by means of evaluation of baseline, perturbation and treatment modules, using Dave or ln[FoldChange] values to calculate PR*i* and PA*i* parameters.

#### 4.4.7. Proteome Recovery and Proteome Activation Index

For each protein it was evaluated its variations (modules) due to perturbation (native vs. pre-EVLP) and/or treatment (pre-EVLP vs. post-EVLP); if the two modules present similar values but opposite sign, the protein recovers its level to the reference (in our case native condition). The extraction of so-called “recovered proteins” was simplified by calculating the Proteome Remodeling index (PR*i*) for each protein using an unbiased procedure. The PR*i* is calculated by the following formula,

$${\mathrm{PR}}_{i}=\frac{M{\left(b-p\right)}_{J}}{M{\left(p-t\right)}_{J}}$$

where J: each identified protein. M(b-p)_J_: Perturbation Module = Difference native vs. pre-EVLP (b: baseline; p: perturbed); differential abundance of specific protein J comparing baseline vs. perturbed (pre-EVLP) conditions. M(p-t)_J_: Treatment Module = Difference pre-EVLP vs. post-EVLP (t:treated); differential abundance of specific protein J comparing perturbed (pre-EVLP) vs. treated (post-EVLP) conditions.

Theoretically, if protein level after treatment remodeled to reference (native) the two modules (perturbation and treatment) should have similar value and opposite sign; then the PR*i* will be negative and close to unit (−1). In our case, we considered proteins with a PR*i* in the range −0.5 to −2.

Also, using a variant of PR index, called Proteome Activated index (PA*i*), it was extracted unchanged proteins between native and pre-EVLP conditions (perturbation module), but activated or inhibited (up- or downregulated, respectively) with treatment (post-EVLP condition). The related formula, based on baseline and treatment modules, is

$${\mathrm{PA}}_{i}=\frac{M{\left(t-b\right)}_{J}}{M{\left(p-t\right)}_{J}}\sim -1$$

M(t − b)_J_: Baseline Module = Difference post-EVLP vs. native (t: treated condition, post-EVLP); differential abundance of specific protein J comparing treated (post-EVLP) vs. baseline conditions.

Differential abundance of each module may be expressed as DAVE value, from MAProMa algorithm or ln[fold change]). However, because in some case the fold-change calculation returns non-sense values (such as n/0 or 0/n), we preferred to use DAVE value to calculate modules. Moreover, MAProMa platform permits the filtration by absolute variation, using DCI algorithm, excluding very low expressed protein (very low spectral count, multiple DCI < |5|) confusingly with noise.

### 4.5. Statistics

Sample size was determined considering a statistical test power of 0.80 and an alpha value of 0.05. Results indicated that a sample size of 15 animals (*n* = 5 per group) would enable to detect a minimum difference in protein expression of 0.35 with an expected standard deviation of 0.15.

To detect outliers, Grubb’s test was applied for each parameter. A probability value <0.05 was considered significant. Baseline characteristics and trends of PAP, Ppeak, glucose, and lactate during ex vivo perfusion were analyzed by means of one-way analysis of variance (ANOVA) followed by Tukey’s multiple comparison test. All analyses were performed using Sigma Stat 11.0 dedicated software (Systat Software Inc., San Jose, CA, USA).

Identified protein were evaluated by LDA (F ratio > 3.4 and a *p*-value < 0.05) [61] and MAProMa (confidence thresholds were set as DAve ≥ |0.2| and a DCI ≥ |10|) platforms.

Finally, proteins extracted by PR*i* and PA*i* algorithms were statistically evaluated by ANOVA and Tukey’s test.

## Supplementary Materials

The supplementary materials are available online.
