# Supplementary material for: Proteome Investigation of Rat Lungs Subjected to Ex Vivo Perfusion (EVLP)
Source: Molecules. 2018 Nov 22;23(12):3061. doi: 10.3390/molecules23123061 (PMC6321151; doi:10.3390/molecules23123061)
Supplement: Supplementary file 1 [file molecules-23-03061-s001.zip › Supplementary Table S1.docx]

**TABLE S1 Baseline characteristics.** Average body weight measured at baseline for each group and mean time intervals of agonal phase, warm ischemia, and cold ischemia for pre-EVLP and post-EVLP groups. One-way ANOVA on ranks followed by Tukey’s multiple comparison test was used to compare three groups one, whereas t-test or Mann–Whitney were performed to compare two groups. No difference was observed among experimental groups, except for warm ischemia (in situ), that was extended in the post-EVLP compared to the pre-EVLP group.

|  |  | **Native** | **pre-EVLP** | **post-EVLP** | **p value** |
| --- | --- | --- | --- | --- | --- |
| **Weight**, g | | 275 [255-307] | 290 [283-301] | 290 [275-299] | 0.696 |
| **Time**, min | |  |  |  |  |
|  | **Agonal phase** | - | 0.34 [1.19-1.32] | 0.43 [0.33-2.22] | 0.548 |
|  | **Warm ischemia** | - | 14.4 ± 0.9 | 19.4 ± 1.9 | 0.045 |
|  | **In situ cold ischemia** | - | 6.6 ± 0.4 | 7.3 ± 0.5 | 0.328 |
|  | **Ex situ cold ischemia** | - | - | 5.8 ± 0.6 | - |
