# Supplementary material for: Proteome Investigation of Rat Lungs Subjected to Ex Vivo Perfusion (EVLP)
Source: Molecules. 2018 Nov 22;23(12):3061. doi: 10.3390/molecules23123061 (PMC6321151; doi:10.3390/molecules23123061)
Supplement: Supplementary file 1 [file molecules-23-03061-s001.zip › Supplementary Table S3.docx]

**TABLE S3 Evaluation of technical and biological repeatability.** Technical replicates (grey background) from duplicate analysis of the same sample; while biological replicates correspond to comparison between samples from the same group. Data are based on SpC. For each comparisons are reported linear regression (R^2^) values that should be close to theoretical values of 1.

**native** R^2^ values: (grey as technical repeatability, blank as biological ones)

| IIIM21 | 0.992 |  |  |  |  |
| --- | --- | --- | --- | --- | --- |
| IIIN23 | 0.946 | 0.988 |  |  |  |
| IIIO25 | 0.941 | 0.919 | 0.982 |  |  |
| IIIP27 | 0.956 | 0.882 | 0.894 | 0.988 |  |
| IIIQ29 | 0.903 | 0.947 | 0.915 | 0.829 | 0.972 |
|  | IIIM21 | IIIN23 | IIIO25 | IIIP27 | IIIQ29 |

**pre-EVLP** R^2^ values: (grey as technical repeatability, blank as biological ones)

| IIF11 | 0.982 |  |  |  |  |
| --- | --- | --- | --- | --- | --- |
| IIG13 | 0.947 | 0.970 |  |  |  |
| IIH15 | 0.906 | 0.864 | 0.979 |  |  |
| III17 | 0.765 | 0.775 | 0.630 | 0.989 |  |
| IIL19 | 0.878 | 0.876 | 0.823 | 0.570 | 0.973 |
|  | IIF11 | IIG13 | IIH15 | III17 | IIL19 |

**post-EVLP** R^2^ values: (grey as technical repeatability, blank as biological ones)

| IA1 | 0.973 |  |  |  |  |
| --- | --- | --- | --- | --- | --- |
| IB3 | 0.925 | 0.968 |  |  |  |
| IC5 | 0.957 | 0.939 | 0.973 |  |  |
| ID7 | 0.937 | 0.949 | 0.942 | 0.980 |  |
| IE9 | 0.462 | 0.422 | 0.474 | 0.440 | 0.984 |
|  | IA1 | IB3 | IC5 | ID7 | IE9 |
