# Supplementary figures and images for: Proteome Investigation of Rat Lungs Subjected to Ex Vivo Perfusion (EVLP)
Source: Molecules. 2018 Nov 22;23(12):3061. doi: 10.3390/molecules23123061 (PMC6321151; doi:10.3390/molecules23123061)

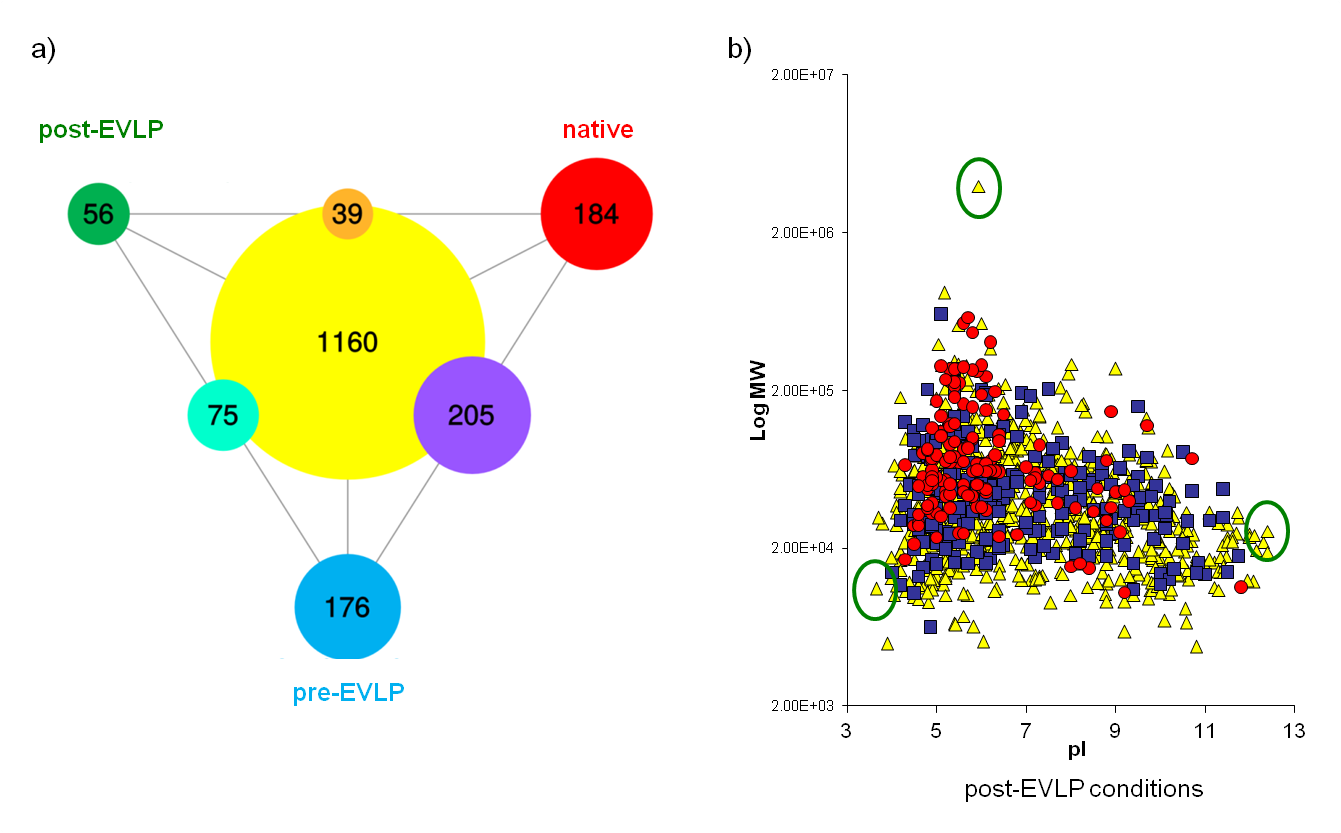

Supplement: Supplementary file 1 [file molecules-23-03061-s001.zip › Supplementary Figure S1.tif]

# Native vs pre-EVLP

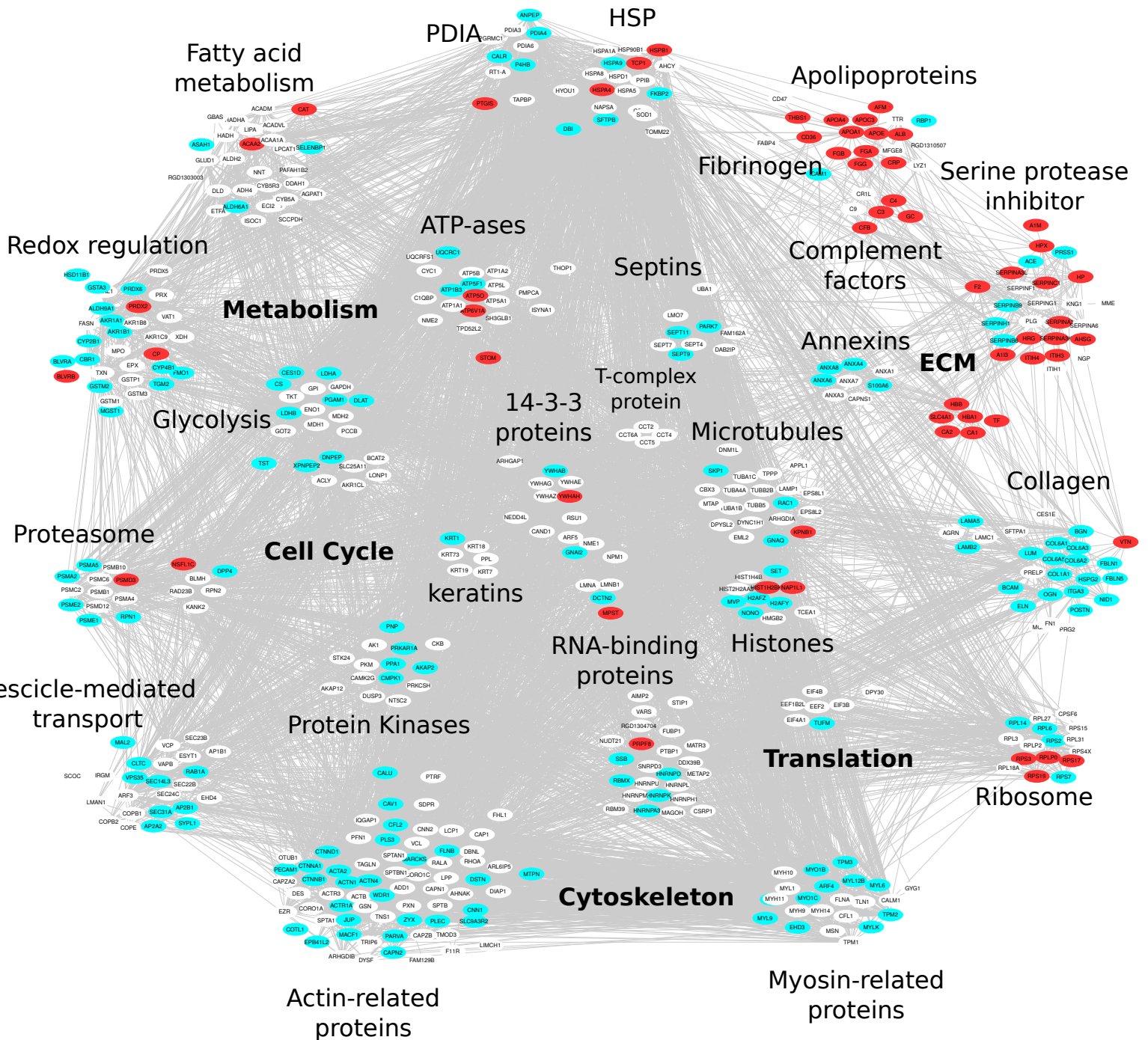

● up-regulated in Native  
● up-regulated in pre-EVLP  
● unchanged

Supplement: Supplementary file 1 [file molecules-23-03061-s001.zip › Supplementary Figure S2a.pdf]

# pre-EVLP vs post-EVLP

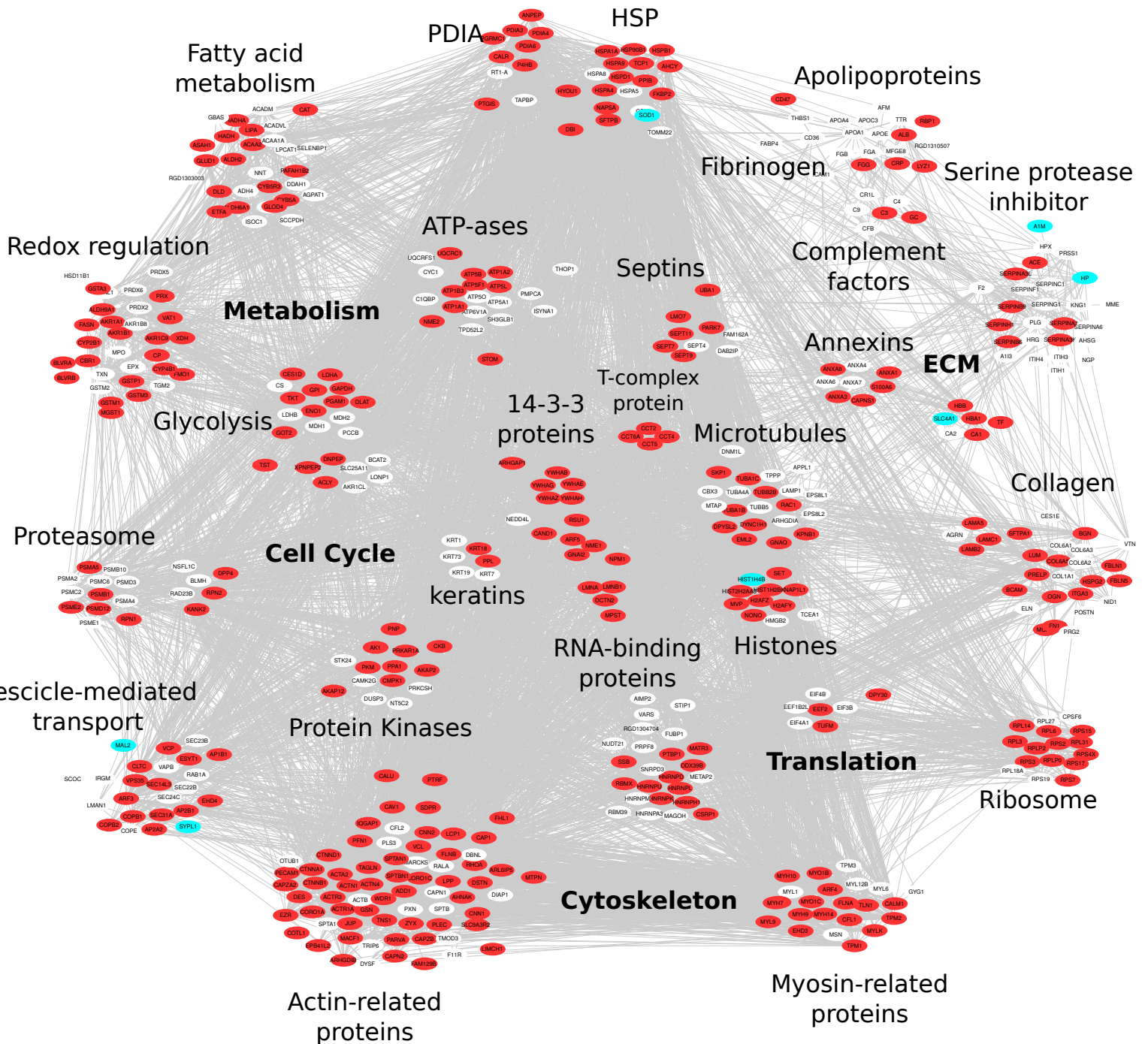

● up-regulated in pre-EVLP  
● up-regulated in post-EVLP  
● unchanged

Supplement: Supplementary file 1 [file molecules-23-03061-s001.zip › Supplementary Figure S2b.pdf]

# Native vs post-EVLP

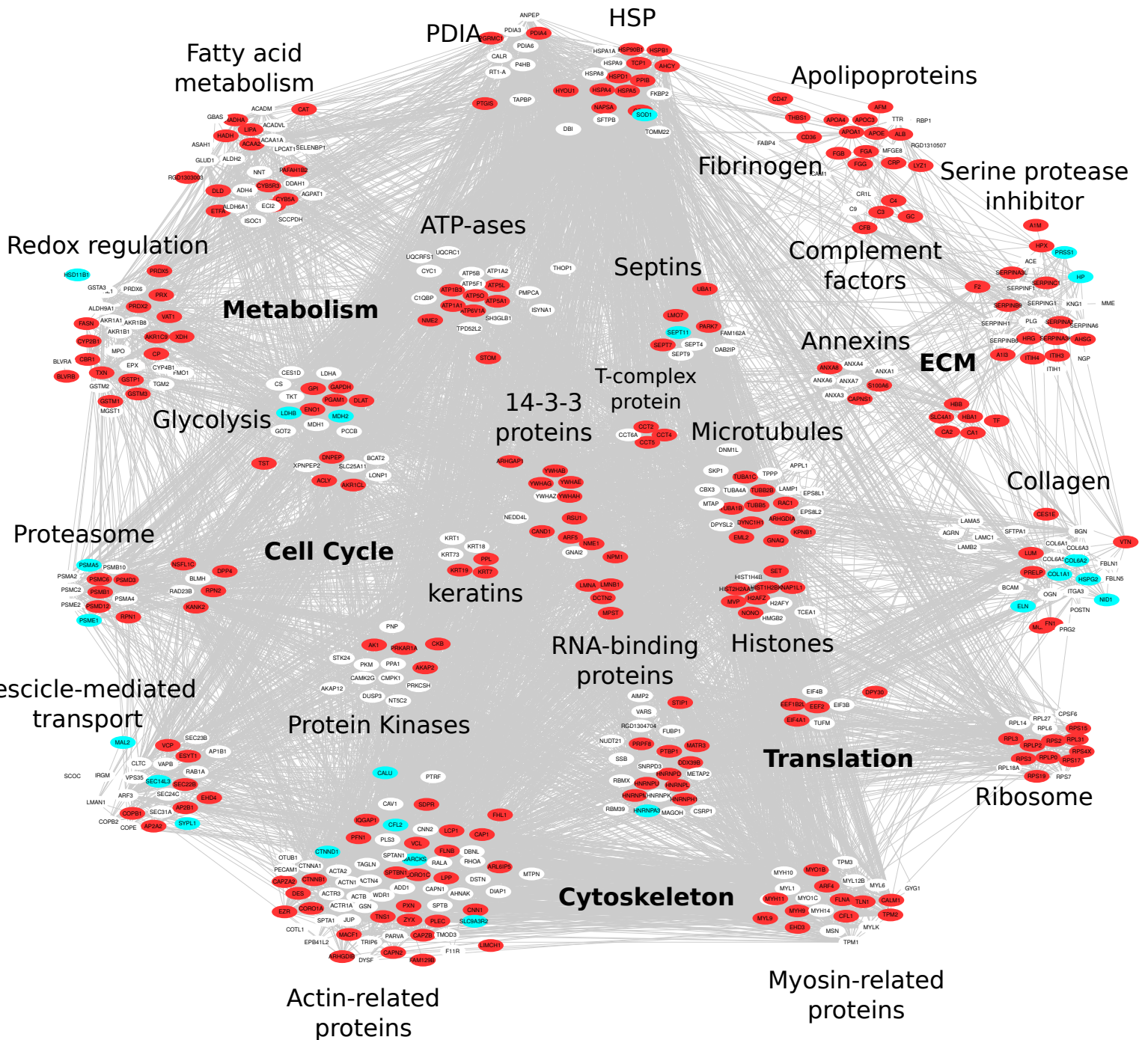

● up-regulated in Native  
● up-regulated in post-EVLP  
● unchanged

Supplement: Supplementary file 1 [file molecules-23-03061-s001.zip › Supplementary Figure S2c.pdf]

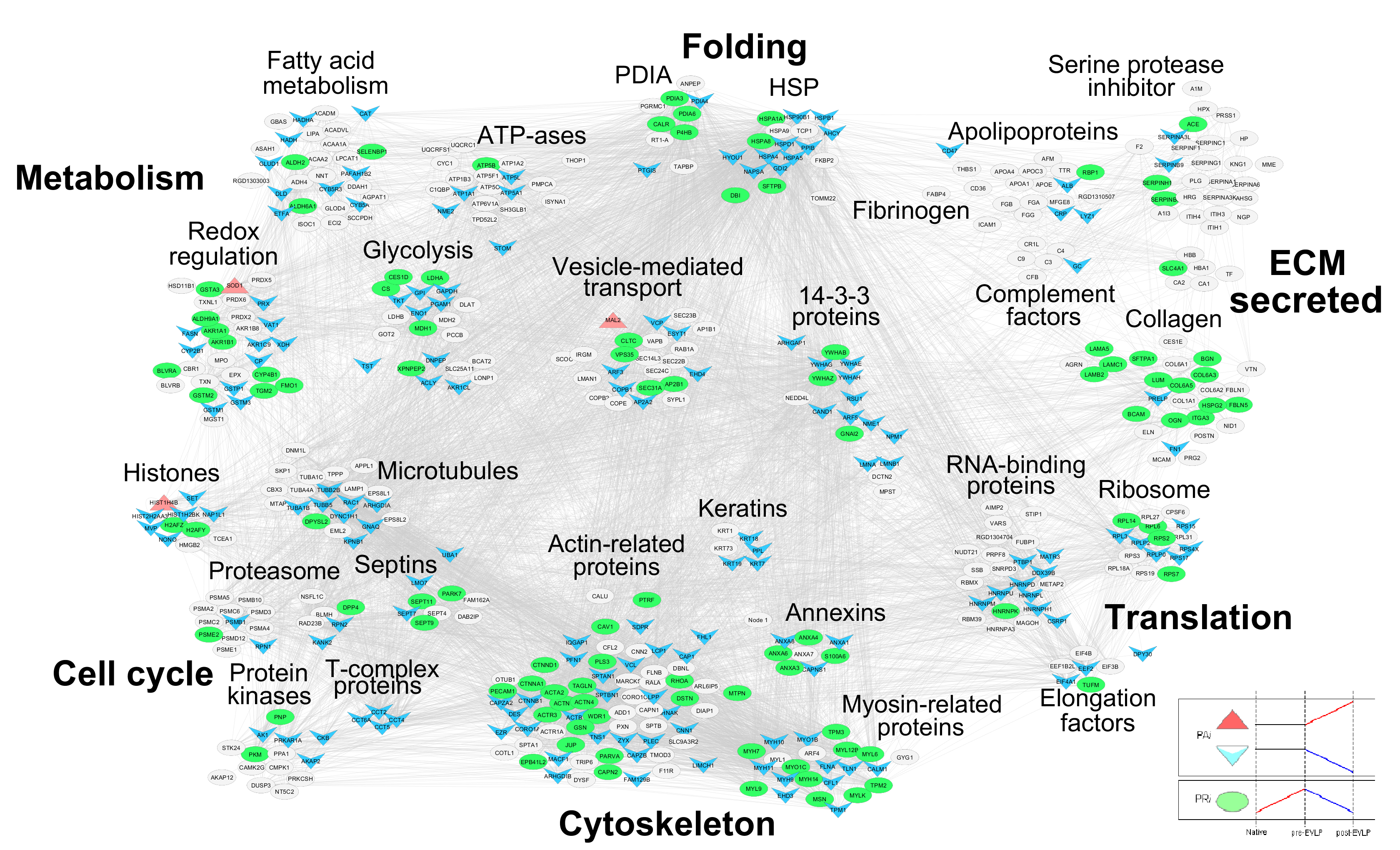

Supplement: Supplementary file 1 [file molecules-23-03061-s001.zip › Supplementary Figure S3.TIF]
