# Supplementary material for: Proteome Investigation of Rat Lungs Subjected to Ex Vivo Perfusion (EVLP)
Source: Molecules. 2018 Nov 22;23(12):3061. doi: 10.3390/molecules23123061 (PMC6321151; doi:10.3390/molecules23123061)
Supplement: Supplementary file 1 [file molecules-23-03061-s001.zip › Figure Captions.doc]

Figure Captions

**FIGURE 1 Experimental pipeline.** Applied workflow for proteomic investigation of frozen and FFPE lung tissues derived from an optimized EVLP rat model. Three distinct conditions (n=5 for each group), native (lungs procured from animals in resting conditions), pre-EVLP (lungs subjected to *in situ* cold flushing and then procured) and post-EVLP (lungs subjected *in situ* cold flushing, procurement and then to *ex vivo* perfusion/ventilation), were analyzed. After protein extraction and digestion, the obtained peptide mixture was analyzed by a nanoLC-MS/MS system in order to allow protein identification and label-free semi-quantitative data-handling, such as repeatability, extraction of differentially expressed and discriminant proteins and systems biology analysis.

**FIGURE 2 Evaluation of lung function during EVLP procedure.** a) PAP, significantly increased (p<0.003), and b) Ppeak, decreased following recruitment manoeuvres (p<0.001). c) Concentration of glucose (from 187±6 mg/dl at 0 min to 174±10 at 180 min, p=0.003) and d) lactate (0.6-0.7 mmol/L at 180 min, p<0.001) significantly changed during reperfusion. PAP, pulmonary artery pressure; Ppeak, peak inspiratory pressure. One-way repeated measures ANOVA; Tukey’s multiple comparison test. p<0.05: a=vs 0 min; b=vs 30 min; c=vs 60 min; d=vs 90 min; e=vs 120 min.

**FIGURE 3 Technical and biological repeatability of proteomic analysis.** Diagrams of correlation coefficient (R2) values of technical and biological replicates. For each R2 value of technical and biological replicates was assigned a color code, and the corresponding ranges associated to a chromatic scale were reported. The technical R2 value (^) was obtained comparing the spectral count (SpC) values from two runs of the same sample. Similarly, plotting the average SpC* values of two samples belonging to the same condition the biological R2 value was calculated. The numerical linear regression values (R2) for each comparison are reported in Supplementary Table S3.

**FIGURE 4 Examples of technical and biological repeatability of proteomic analysis.** a) Typical technical repeatability (IIIQ29 sample, native group). b) Biological repeatability between two native samples (IIIM21 and IIIN23). c) Biological repeatability of IE9 sample compared to ID7 (post-EVLP samples). d) Biological repeatability of IE9 sample compared to ID7 without the scattered proteins (mainly plasma proteins). SpC*: Average spectral count value.

**FIGURE 5 Hierarchical clustering obtained by Linear Discriminant Analysis (LDA).** Using the average SpC values from all conditions, the post-EVLP samples were correctly segregated and 451 descriptor proteins by LDA were identified. Of note, IE9 sample, although remaining within the correct subgroup, is localized on a separate branch. The complete list of descriptor proteins extracted by LDA is reported in Supplementary Table S4.

**FIGURE 6 Main pathways involved in the proteome remodelling induced by the EVLP procedure.** Enriched sub-networks based on recovered and activated proteins after ANOVA (<0.05) and Tukey test (<0.05; *<0.08). These statistically significant proteins were previously extracted using, an unbiased procedure that evaluated simultaneously the changes in the proteome in the three conditions: native, pre-EVLP and post-EVLP. Recovered proteins, marked as a green/circle, were extracted using the Proteome Remodelling index (PR*i*). While, activated proteins marked as red/triangle and blue/arrow for up- and down-regulated in post-EVLP, respectively, were extracted using Proteome Activated index (PA*i*). The application of PR*i* and PA*i* filters highlights an enrichment in a) vesicle-mediated transport, myosin- and actin-related proteins, belonging to cytoskeleton and b) redox regulation, belonging to metabolism. The blank nodes identify the protein descriptors resulted outside the applied PR*i* and PA*i* filters. c) summary of considered trends of remodelled proteins. The complete lists of PR*i* and PA*i* extracted proteins are reported in Supplementary Tables S5-S6.

**FIGURE 7 Comparison between frozen and FFPE pre-EVLP lung lobes.** a) protein overlapping between frozen and FFPE in three pre-EVLP analyzed samples (IIL11, IIG13 and IIH15). b) Plot of frozen vs FFPE samples using normalized average spectral count (SpC*) values. c) Distribution of proteins between the frozen and FFPE according to their molecular function using Protein ANalysis THrough Evolutionary Relationships (Panther) classification system. For each molecular function, reported as percentage of proteins for the two tissues (blue/solid colour for frozen and blank for FFPE datasets), the difference frozen vs FFPE was <0.9 %.
