# Supplementary material for: Proteome Investigation of Rat Lungs Subjected to Ex Vivo Perfusion (EVLP)
Source: Molecules. 2018 Nov 22;23(12):3061. doi: 10.3390/molecules23123061 (PMC6321151; doi:10.3390/molecules23123061)
Supplement: Supplementary file 1 [file molecules-23-03061-s001.zip › Supplementary Figure and Table Captions.doc]

**Supplementary Figure Captions**

**FIGURE S1. Shared proteins and map of frozen lung lobes.** a) Proportional Venn diagram of protein distribution in the three examined conditions: native, pre-EVLP and post-EVLP. b) 2D virtual map of identified proteins, according to their Isoelectric point (pI) and the logarithm of Molecular Weight (Log MW);a color/shape code was assigned for each protein according to the confidence of SEQUEST score values (yellow/triangle ≤ 15, blue/square 15-35 and red/circle ≥ 35). Of note, using gel-free proteomics approach it was possible to characterize proteins with extreme theoretical MW and/or pI values, highlighted with a circle, such as titin (Accession: 564302435, MW: 3941808 Da) phosphatidylethanolamine binding protein (Accession: 406294, MW: 4934), ribosomal protein L18 (Accession: 89573867, pI: 12.40) and acidic leucine-rich nuclear phosphoprotein 32 family member B (Accession: 18777770, pI: 3.70). The corresponding list of all identified proteins is reported in Supplementary Table S2.

**FIGURE S2. Protein-Protein interaction networks of proteins extracted by LDA and DEP.** Metabolic networks reporting both differentially expressed proteins (DEP) and descriptors (LDA). For each protein (node) was assigned a color code that indicates the DAve value calculated: red and blue codes indicate that the protein is up-regulated in the first and second conditions, respectively. The blank nodes identify the undifferentiated proteins with Dave and DCI below fixed thresholds (|0.2| and |10|, respectively). Three comparisons are reported: a) native vs pre-EVLP, b) pre-EVLP vs post-EVLP and c) native vs post-EVLP. The complete protein list with the corresponding DAve and DCI values and the calculated F-ratios and p-values is reported in Supplementary Table S4.

**FIGURE S3. Protein-Protein interaction networks of proteins extracted by PA*i* and PR*i*.** Metabolic networks reporting both recovered and activated proteins. For each protein (node) was assigned a color/shape code which indicates its trend in the three conditions (native, pre-EVLP and post-EVLP): recovered proteins, marked as a green/circle; activated proteins marked as red/triangle and blue/arrow for up- and down-regulated in post-EVLP, respectively. The blank nodes identify the protein descriptors resulted outside the applied PR*i* and PA*i* filters. The detailed lists of PR*i* and PA*i* extracted proteins are reported in Supplementary Tables S5 and S6.

**Supplementary Table Captions**

**TABLE S1 Baseline characteristics.** Average body weight measured at baseline for each group and mean time intervals of agonal phase, warm ischemia, and cold ischemia for pre-EVLP and post-EVLP groups. One-way ANOVA on ranks followed by Tukey’s multiple comparison test was used to compare three groups one, whereas t-test or Mann–Whitney were performed to compare two groups. No difference was observed among experimental groups, except for warm ischemia (*in situ*), that was extended in the post-EVLP compared to the pre-EVLP group.

**TABLE S2. Identified Proteins in frozen samples.** Lists of the distinct proteins identified in frozen tissue lung lobes stratified in the three considered conditions: native, pre-EVLP and post-EVLP. For each protein are reported: NCBI Accession (GI number), Uniprot Accession, Gene name, Reference, pI and MW, Frequency, SpC and Score. Frequency indicates how many times a given protein has been identified under each condition examined. The asterisk next to SpC and Score indicates that the average values are given for each protein of the same condition.

**TABLE S3. Evaluation of technical and biological repeatability.** Technical replicates (grey background) from duplicate analysis of the same sample; while biological replicates correspond to comparison between samples from different animals of the same group. Data are based on SpC values. For each comparison are reported R^2^ values that should be close to theoretical values of 1.

**TABLE S4. DEP and LDA proteins.** Complete list of the differentially expressed proteins among the three conditions examined and the descriptors identified by LDA. Three comparison are considered; native vs pre-EVLP, pre-EVLP vs post-EVLP and native vs post-EVLP. For each protein are reported: GI Accession number, Gene name, Reference, pI, MW, the average SpC value, F ration and P-value calculated by LDA, DAve and DCI value calculated by MAProMa software. Positive values for DAve and DCI indicate that the protein is more abundant in the first condition, negative values in the second one. Differentially expressed proteins (DEP) that exceed DAve and DCI applied thresholds are highlighted in yellow. For further details regarding the meaning and the confidence range applied to DAve, DCI and LDA see Materials and Methods section. Value (DAve and/or DCI; F Ratio and/or p value) is not reported when it has not passed the fixed threshold.

**TABLE S5**. **Proteome remodelling.** List of the remodelled proteins extracted by PR*i* algorithm. Two comparison are considered; native vs pre-EVLP and pre-EVLP vs post-EVLP. Positive values for DAve and DCI indicate that the protein is more abundant in the first condition, negative values in the second one. Proteins that pass the filters are reported in bold. When ANOVA p-value resulted > 0.0.5 Tukey’s test was not calculated. Accession: GI number SpC*: average spectral count. For further details regarding the meaning and the confidence range applied to DAve and DCI, LDA, PR*i* and ANOVA and Tukey’s test see Materials and Methods section.

**TABLE S6. Proteome Activated.** List of the activated proteins extracted by PA*i* algorithm. Two comparison are considered; native vs post-EVLP and post-EVLP vs pre-EVLP. Positive values for DAve and DCI indicate that the protein is more abundant in the first condition, negative values in the second one. Proteins that pass the filters are reported in bold. When ANOVA p-value resulted > 0.0.5 Tukey’s test was not calculated. Accession: GI number; SpC*: average spectral count. For further details regarding the meaning and the confidence range applied to DAve and DCI, LDA, PA*i* and ANOVA and Tukey’s test see Materials and Methods section.

**TABLE S7.** **Frozen and FFPE protein profiles of pre-EVLP condition.** Lists of the distinct proteins identified in FFPE and frozen samples from pre-EVLP tissue lobes. Frequency indicates how many times a given protein has been identified under each condition examined. Accession: GI number; SpC*: average spectral count, absolute and normalized (norm); Score*: average score.
